# A cluster-randomized trial of task shifting and blood pressure control in Ghana: study protocol

**DOI:** 10.1186/1748-5908-9-73

**Published:** 2014-06-12

**Authors:** Gbenga Ogedegbe, Jacob Plange-Rhule, Joyce Gyamfi, William Chaplin, Michael Ntim, Kingsley Apusiga, Kiran Khurshid, Richard Cooper

**Affiliations:** 1 Center for Healthful Behavior Change, Division of Health & Behavior, Department of Population Health, New York University School of Medicine, 550 1st Avenue, New York, NY 10016, USA; 2 School of Medical Sciences, Kwame Nkrumah University of Science and Technology, Accra Road, Kumasi, Ghana; 3 Stritch School of Medicine, Loyola Chicago Medical Center, 2160 South 1st Avenue, Maywood, IL 60153, USA; 4 Department of Psychology, Saint Johns University, 8000 Utopia Pkwy, Queens, New York, NY 11439, USA

**Keywords:** Hypertension, Cluster randomized controlled trial, Task shifting, Blood pressure control, Community health centers, Community health nurses, Ghana

## Abstract

**Background:**

Countries in sub-Saharan Africa (SSA) are experiencing an epidemic of cardiovascular disease (CVD) propelled by rapidly increasing rates of hypertension. Barriers to hypertension control in SSA include poor access to care and high out-of-pocket costs. Although SSA bears 24% of the global disease burden, it has only 3% of the global health workforce. Given such limited resources, cost-effective strategies, such as task shifting, are needed to mitigate the rising CVD epidemic in SSA. Ghana, a country in SSA with an established community health worker program integrated within a national health insurance scheme provides an ideal platform to evaluate implementation of the World Health Organization (WHO) task-shifting strategy. This study will evaluate the comparative effectiveness of the implementation of the WHO Package targeted at CV risk assessment versus provision of health insurance coverage, on blood pressure (BP) reduction.

**Methods:**

Using a cluster randomized design, 32 community health centers (CHCs) and district hospitals in Ghana will be randomized to either the intervention group (16 CHCs) or the control group (16 CHCs). A total of 640 patients with uncomplicated hypertension (BP 140–179/90–99 mm Hg and absence of target organ damage) will be enrolled in this study (20 patients per CHC). The intervention consists of WHO Package of CV risk assessment, patient education, initiation and titration of antihypertensive medications, behavioral counseling on lifestyle behaviors, and medication adherence every three months for 12 months. The primary outcome is the mean change in systolic BP from baseline to 12 months. The secondary outcomes are rates of BP control at 12 months; levels of physical activity, percent change in weight, and dietary intake of fruits and vegetables at 12 months; and sustainability of intervention effects at 24 months. All outcomes will be assessed at baseline, six months and 12 months. Trained community health nurses will deliver the intervention as part of Ghana’s community-based health planning and services (CHPS) program.

**Discussion:**

Findings from this study will provide policy makers and other stakeholders needed information to recommend scalable and cost-effective policy with respect to comprehensive CV risk reduction and hypertension control in resource-poor settings.

**Trial registration:**

NCT01802372.

## Background

Ghana and other countries in sub-Saharan Africa (SSA) are experiencing an epidemic of CVD propelled by rapidly increasing rates of hypertension [1]. According to the World Health Organization (WHO), 75% of deaths in SSA will be attributable to hypertension by the year 2020 [2]. Similar to most countries in SSA, Ghana is undergoing a rapid epidemiological transition [3], such that the last decade has seen major causes of death shift from solely infectious diseases to a combination of communicable and non-communicable diseases (NCDs) [4]. Hypertension is the second leading cause of morbidity in Ghana among adults older than 45 years [5]. In a systematic review of eleven surveys conducted in Ghana, the prevalence of hypertension ranged between 19.3% in rural areas to 54.6% in urban areas [6], and the levels of hypertension detection, treatment, and control were abysmally low in six studies where such data was reported—blood pressure (BP) control rates were between 1.7% to 12.7%. Findings from a population-based study by Cappuccio et al. showed that in the Ashanti Region of Ghana, more than one in four adults have hypertension [7]. Such high prevalence and the abysmally low rates of BP control is largely responsible for the increasing burden of CVD mortality, particularly stroke, in Ghana. For example, in Accra, CVD rose from being the seventh and tenth cause of death in 1953 and 1966, to the number one cause of death in 1991 and 2001, respectively [8].

Thus, interventions targeted at BP control are vital to reducing hypertension-related morbidity and mortality [9,10]. However, socioeconomic barriers, lack of insurance coverage, uncoordinated care, and shortage of physicians limit the capacity of SSA countries to implement CVD prevention at the primary care level [9,11-15]. For example, although SSA harbors 11% of the world’s population and bears over 24% of global disease burden, it has only 3% of the global health workforce, and spends less than 1% of the world’s financial resources on health [16]. Given such limited resources, cost-effective systems-level strategies are urgently needed to mitigate the rising epidemic of CVD in SSA [9,17]. To date however, little is known about effective interventions targeted at BP control in SSA [18], especially notable is the lack of interventions targeted at systems-level barriers to optimal hypertension control on a wide-scale level in the region.

Task shifting of healthcare duties from physicians to non-physician healthcare providers (NPHCPs) at the primary care level may mitigate the barriers to optimal hypertension control in SSA. Task shifting, which is defined as the rational distribution of tasks among health workforce teams, is especially useful in low-resource settings facing healthcare human resource crisis [19], such as SSA countries. Given the limited healthcare resources and acute shortage of physicians in SSA, cost-effective and affordable approaches are urgently needed to meet the global challenge of the CVD epidemic in the region [20]. In this context, shifting the tasks of CV risk assessment and management of uncomplicated CV risk factors, such as hypertension and diabetes, from physicians to non-physician healthcare providers such as nurses is a viable and potentially cost-effective strategy. Task shifting, has been proposed as a potential solution to not only solving the acute shortage of physicians in SSA but as a viable method to implement primary and secondary prevention at the primary care level [21]. The benefits of task shifting in addressing the HIV/AIDS epidemic include: increasing access to life-saving treatment; improving health-system efficiency; and enhancing the role of the community [21]. Fortunately, primary and secondary prevention of cardiovascular (CV) risk factors such as hypertension, often involves lifestyle counselling, adoption of self-management skills, and in some cases implementation of well-protocolized medication therapy that can often be instituted by NPHCPs instead of the costlier use of physicians [22]. There is growing evidence that patients with hypertension can be cared for by NPHCPs, who provide knowledge of the beneficial effects of a healthy lifestyle to patients almost as often as physicians [20,22-24].

The conceptual framework for this study is based on three viable, and potentially cost-effective strategies for mitigating systems-level barriers to optimal hypertension control in resource-poor settings: shifting integrated interventions for CV risk reduction and hypertension control to primary care; task shifting of primary care duties from physicians to non-physician healthcare providers such as nurses for management of chronic diseases [20,21,25]; and provision of health insurance coverage to patients, who otherwise cannot afford the high out-of-pocket payments and medications [26]. In this regard, the World Health Organization (WHO) has developed a *Package of Essential Noncommunicable Disease Intervention for Primary Care (WHO PEN)*[25], which includes clinical decision support for management of CVD via easy-to-follow algorithms; lifestyle counseling; and drug treatment protocols delivered by community health nurses [25]. Its efficacy at improving BP control is well proven in low- and middle-income countries (LMICs) and is considered a ‘Best Buy’ [10,11,24]. However implementation of this program is almost non-existent in SSA. If these interventions do not find a place at the primary care level in SSA countries, their impact on public health as well as the CVD epidemic would be negligible. Two key aspects of the healthcare system in Ghana, makes it a unique country to evaluate the implementation of WHO PEN. First, a key aspect of the healthcare system in Ghana is the widespread implementation of Community-based Health Planning and Services (CHPS) program as a strategy to use community health nurses to deliver primary care services at community health centers (CHCs) across the country [27]. The community health nurse is the fulcrum of CHPS, and they play a vital role in delivery of primary health care at both the household and community level. The CHPS program thus provides an opportunity for implementing task-shifting strategy targeted at chronic diseases management and may serve as a model for other LMICs. Second, in 2003, Ghana instituted a National Health Insurance Scheme (NHIS) that provides access to primary care, medical consultations, laboratory tests, and medications at low cost [26]. Thus, availability of NHIS for uninsured patients, and widespread implementation of CHPS, as a strategy for delivery of primary care services, presents a unique opportunity to evaluate the impact of both strategies on hypertension control in Ghana.

### Study aims and hypothesis

This study will evaluate, among 640 hypertensive patients who receive care in CHCs in the Ashanti Region of Ghana, the comparative effectiveness of the implementation of the WHO PEN program targeted at CV risk assessment and hypertension control (intervention group), versus provision of health insurance coverage (control group), on BP reduction. The secondary aims are to evaluate the effect of the WHO PEN program vs. provision of health insurance coverage on BP control, lifestyle behaviors, and the sustainability of the intervention effects one year after the trial is completed. We hypothesize that patients randomized to the intervention group (IG) compared to those to control group (CG), will have a greater reduction in systolic BP at 12 months, a higher proportion of BP control at 12 months, higher levels of physical activity; weight loss, and intake of fruits and vegetables, and they will maintain higher BP control rates and greater BP reduction at 24 months (one year after completion of the trial).

## Methods

### Study design

**Figure 1:**
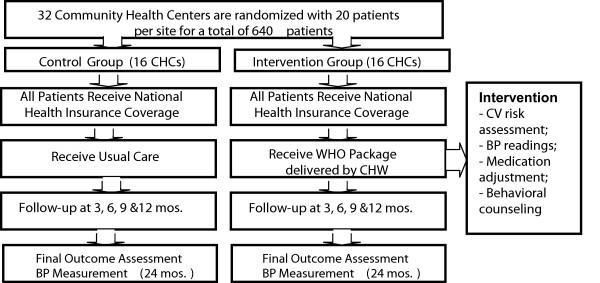
Overview of study design.

As shown in Figure 1, using a cluster randomized trial design, 32 CHCs in the Ashanti Region of Ghana will be randomly assigned to either the intervention group (16 CHCs) or the control group (16 CHCs). A total of 640 patients with uncomplicated hypertension (systolic BP 140 – 179 mm Hg or diastolic BP 90 – 99 mm Hg and absence of target organ damage) will be enrolled in this study with about 20 patients per CHC. Study outcomes will be assessed every three months for one year, and sustainability of intervention effects will be evaluated at 24 months (one year after the trial is completed). Patients at the CHCs randomized to the control group will receive health insurance coverage in addition to usual care. Patients at the CHCs randomized to the intervention group will also receive health insurance coverage plus the WHO PEN package that will be delivered by trained community health nurses as indicated in the treatment protocol. The WHO PEN protocol consists of four basic steps: inquiry about patient’s health history (heart attack, angina, stroke, transient ischemic attack, diabetes, and lifestyle behaviors); physical and laboratory examination (including BP measurements, anthropometrics, urine dip stick, fasting glucose, and plasma cholesterol); estimation of patient’s CV risk based on the WHO risk charts (low, medium, or high); and subsequent initiation of drug therapy and lifestyle counselling during follow-up visits [25]. Depending on the patient’s CV risk, the treatment decisions include either an immediate referral to a specialist in the case of patents with high CV risk and (BP ≥180/100); or lifestyle counselling on diet, physical activity, and tobacco cessation; prescription of an antihypertensive medication; and follow-up with a provider. The intervention will occur every three months for 12 months during scheduled study visits.

### Study setting and participants

Participating CHCs were selected from areas that are geographically distant from one another with equal urban/rural mix in the Ashanti Region, which has about 170 community health centers. Potentially eligible patients will be identified from the CHCs through physician referrals and the community health nurses. The study was approved by the Institutional Review Board of New York University School of Medicine and the Committee for Human Research, Publications and Ethics at Kwame Nkrumah University of Science and Technology, Kumasi, Ghana.

### Health center recruitment and randomization

Eligible CHCs must have at least one community health nurse employed in the CHPS program; be a certified NHIS provider; and have basic capability for blood tests [28]. Eligible CHCs were identified with the help of the Kumasi Metropolitan Director of Health for the Ghana Health Services, who oversees all the health centers in the region. A total of 32 CHCs will be randomized in cohorts of eight CHCs each for a total of four cohorts. When recruitment for each cohort is completed, the CHCs are randomized to the intervention or control group in a 1:1 ratio. The sequence of randomization is generated by the study statistician, and kept in sealed opaque envelopes away from the study sites in accordance with CONSORT guidelines [29]. Once the sites have been recruited, they are informed of their randomization group by telephone. Because of the nature of the intervention, it is impossible to blind the patients, community health nurses, and the study coordinators to the group assignment. However, the salient dimension that the study coordinator could possibly affect is the primary outcome of BP measurements. To mitigate this bias, we will use an automated BP device, which prevents the study coordinator from influencing in any manner the BP readings.

### Staff recruitment and training

Nurses selected for the study were active workers at the various health centers. Training procedures was centralized in Ghana and occurred before the start of the project and then every six months during patient recruitment. All study staff received training on the study protocol, including the WHO CVD risk assessment package, techniques for collecting anthropometric data, BP measurements, questionnaire administration, blood draw, urine collection, and processing techniques, which were standardized between health facilities. Study nurses received additional training in behavioral counselling techniques using motivational interviewing (MI). Project coordinators and nurses received training in proper procedures for obtaining informed consent from all study participants and study data collection. The study nurses are responsible for screening, and referring patients with complicated hypertension to a specialist; assisting with data collection as necessary, and conducting the MI counselling sessions with patients. All field staff involved in data collection were trained and certified before data collection began.

### Participant recruitment

Potentially eligible patients are identified from the CHCs through physician referrals and the community health nurses. Each CHC will recruit 20 consecutive patients who meet the following eligibility criteria: registered to receive care at the CHC; adults age 40 years and older; have BP 140 – 179/90 – 99 mm Hg, are not receiving treatment for hypertension; and are able to provide informed consent. Given low literacy levels, consent will be provided both verbally and in written form. Patients will be excluded if they have a diagnosis of diabetes, coronary artery disease, transient ischemic attacks, stroke, heart failure, angina; BP ≥ 180/100 mm Hg; positive urine dipstick for protein; are pregnant; and unable to comply with the follow-up requirements or provide informed consent. Patients found on examination to have a history of transient ischemic attacks (TIAs), stroke, heart failure, diabetes, angina, claudication, and BP ≥ 180/100 mm Hg will be referred to the district hospitals for further management.

### Description of the intervention

#### Provision of health insurance coverage to patients in both arms of the study

Patients in both arms of the study will receive health insurance coverage for 12 months. In 2003, Ghana instituted a National Health Insurance Scheme (NHIS) that provides access to primary care, medical consultations, laboratory tests, and medications at low cost [29]. The rationale for the provision of health insurance for all patients is to eliminate socioeconomic and systems-level barriers to optimal hypertension control that is well documented in SSA [28].

### Intervention Group (IG)

In addition to NHIS coverage, patients at the CHCs randomized to the IG will receive the WHO PEN [25,30], which includes clinical decision support for management of CVD via easy-to-follow algorithms; lifestyle counselling; and drug treatment protocols delivered by trained community health nurses as part of the widespread CHPS program in Ghana. Linking the services of the community health nurses to CHPS allows us to assess the scalability of the WHO strategy for CV risk assessment and optimal hypertension control in Ghana. The community health nurses will deliver the protocol for the WHO Package every three months for one year in five study visits [10]. Briefly, once patients’ eligibility is confirmed, trained community health nurses at the intervention sites will take the patients’ medical history. Next, they will measure the patients’ weight, height, waist circumference, and BP with an automated device. The community health nurse then performs laboratory tests using point-of-care testing to assess fasting blood glucose, cholesterol levels, and urine dipstick for proteinuria. The information obtained from the medical history, physical examination, and laboratory tests are then used by the nurse to assess patients’ CV risk with the aid of the well validated WHO risk charts that are contextualized for each region [30]. Once the patient’s CV risk is determined (low, medium or high), those with high CV risk will be referred to the district hospital for further management. The community health nurse will initiate the treatment protocol for patients who are at low or medium risk according to their BP level using any one of the four major antihypertensive medications—bendrofluazide, a diuretic; amlodipine, a calcium channel blocker (CCB); Lisinopril, an angiotensin-converting enzyme (ACE) inhibitor; and bisoprolol, a beta-blocker (BB). Those with stage one hypertension will receive 2.5 mg of bendrofluazide or 5 mg Amlodipine. Thus, those patients with stage two hypertension (BP160 – 179/90 – 99 mm Hg) will receive combination therapy with two antihypertensive medications at low dose using either a diuretic plus ACE inhibitor; or a CCB plus ACE inhibitor; or a diuretic plus a BB; or a CCB plus a BB following JNC-7 guidelines [31]. In either case, medication titration will be initiated at subsequent study visits every three months, if BP control is not achieved [10]. Medications will be provided to patients for free and they will not be required to pay for their clinic visits. In addition, all patients will receive information on the expected side effects of their medications and asked to return to the health center if they experience unexplained side effects. Finally, once the medications have been prescribed, patients will be counselled on lifestyle behaviors targeted at addressing barriers to medication adherence, physical activity, dietary intake of fruits and vegetables, and weight loss using motivational interviewing techniques. The community health nurses will be trained in delivery of the WHO Package for CV risk assessment and in lifestyle counselling similar to that used in the WHO-sponsored trial by Mendis et al. [10].

### Control group (CG)

In addition to NHIS coverage, patients at the CHCs randomized to the CG will receive usual care as determined by their providers. They will be given information materials on hypertension and patient education about the causes of hypertension and its treatment. Similar to the IG, these patients will also have a total of five study visits for 12 months (one every three months), during each visit their BP will be assessed and the levels of lifestyle behaviors including physical activity, dietary intake of fruits and vegetables, and their weight will also be assessed. These patients will not receive the WHO Package but will be encouraged to follow up with their healthcare providers for the treatment of their hypertension.

### Procedures for data collection and study measures

#### Physiological/laboratory measures

##### Blood pressure measurements

Three BP readings will be taken by trained study coordinators using an automated BP monitor [32] with the patient seated comfortably for five minutes prior to the measurements, following the American Heart Association guidelines [33]. The same procedure will be followed at six- and 12-month study visits. The average of the three BP readings will be used as the measure for each visit. Uncontrolled BP is defined as average clinic SBP ≥140 mm Hg or DBP ≥90 mmHg following JNC-7 guidelines [31].

Height and weight will be measured without shoes using a tape rule and a validated digital scale respectively. All measurements will be recorded to the nearest 0.1 cm and 0.1 kg respectively. These data will be used to assess patients’ body mass index (BMI), which will serve as a measure of weight loss.

Fasting blood specimen will be collected after an overnight fast. The purpose of blood collection is to allow us to evaluate indicators of CV risk (lipid profile, and fasting glucose). This will be achieved by point of care testing. The results of these data will be used to assess patients’ CV risk score.

### Self-report measures

The study data collection tools will be administered in English because most participants speak and understand English. However, trained study coordinators fluent in Twi, will translate the data collection instruments to illiterate participants on as needed basis. Twi is the dialect spoken in Kumasi, the Ashanti Region where this study is being undertaken.

### Patient demographics

Socio-demographic data will be used to describe the cohort and examine effects of these factors on the study outcomes. Variables include age, gender, household income, education level, marital status, and employment status.

### Medication adherence

Adherence to prescribed antihypertensive medications will be assessed using the widely used and validated four-item scale developed by Morisky that specifically addresses adherence to prescribed medication regimen [34]. Data on medication adherence will allow us to assess the potential effect of adherence on BP control.

### Dietary intake

Dietary intake will be assessed using a 24-hour food recall, a validated, self-administered instrument designed to measure energy intake (kcal) and macronutrient intake during the previous 24 hours [35]. We will use a validated system for portion size estimation which has been utilized in multiple countries in SSA [36].

### Physical activity

Physical activity will be assessed with the Global Physical Activity Questionnaire, developed by the WHO for physical activity surveillance in LMIC countries [37].

### Chart extraction

Information extracted from the medical records will include medications and their dosages, BP measurements from clinic visits, and medical comorbidity.

### Outcome measures

The primary outcome is the mean change in systolic BP from baseline to 12 months. The secondary outcomes include proportion of patients with adequate BP control at 12 months; levels of physical activity, percent change in weight, and dietary intake of fruits and vegetables at 12 months; and BP control rates at 24 months. BP control is defined as BP < 140/90 mmHg following JNC-7 guidelines [31]. BP readings will be assessed with validated automated BP device. All study outcomes will be assessed at baseline, six months, and 12 months.

### Sample size and power analysis

The study is a two-arm cluster randomized trial with the health center as the unit of randomization. Thus, there are two important sample size estimates, N the number of patients and K, the number of clinics, with the patients nested within the K clinics. The sample size calculations is based on a two-tailed alpha level of p = 0.05, and power at 0.80. The effect size is based on a greater reduction in SBP of 5 mm Hg in the IG compared to the CG. A standard deviation of SBP of 15 mm Hg [10], which yields a standardized effect size of BP change of d = 0.33 and an ICC of 0.06 consistent with the data from the WHO-sponsored Mendis et. al trial in Nigeria [10] was used to estimate the sample size. Based on this information the combinations of clinics and patients/clinic in Table 1 satisfies our desire for power = 0.80. Table 1 illustrates the flexibility in sample size estimates with respect to both recruitment and attrition. For example, if the recruitment goal is an average of 30 patients in each clinic, but only an average of 20 per site was achieved, it means six more clinics (26 + 6 = 32) will need to be randomized to maintain a statistical power of 0.80. Likewise, if an attrition rate of 25% among the 20 patients was experiences, then four more clinics (32 + 4 = 36) will need to be recruited in order to maintain the same power level with an average of 15 completers/clinic.

**Table 1:** Sample size

**Number of clinics**	**Number of patients**	Sample size*
26	30	780
32	20	640
36	15	540
44	10	440

*Total sample is the product of clinics and patients/clinic.

### Statistical methods

#### Analysis for primary hypotheses

We hypothesize that patients in the intervention group will have greater systolic BP reduction than those in the control group at 12 months. This analysis will be accomplished with a multilevel MANOVA (unstructured covariance matrix across three time points baseline, six, and 12 months). This analysis will have one within-person factor—time (baseline, six-month, and 12-month follow-up) and one primary between-patient factor (randomization group). The outcome measures in this analysis will be systolic BP. Additionally the patients will be nested within clinics creating a three-level analytic model (observations nested within patients nested within clinics). Multilevel modeling software (SAS, Version 9, PROC MIXED) will be used to compute full-information maximum likelihood (FIML) estimates of the model parameters [38]. The PROC MIXED procedure will use an error structure that allows for the possibility of group differences in the error variances at follow-up; and the serial correlations of baseline BP with six- and 12-month BP. The primary test concerns the Group X Time interaction, and the resulting F-test will provide the primary ‘intent-to-treat’ test of the hypothesis. If this is statistically significant at the two-tailed α = 0.05 level, for ease of interpretation, we will estimate and report the magnitude of the treatment effect, with 95% CI for SBP. Ideally, the randomization of participants to treatment arm and the absence of significant selection and/or attrition biases will obviate the need for any covariates in the analysis. However, in the event that the above-described probit analyses indicate one or more sources of potential bias, the predicted values of those analyses will be included as covariates in the MANOVA (including their interactions with the within-person factors).

### Analysis for secondary hypotheses

#### Secondary hypothesis one: patients in the intervention group will have higher BP control rate than those in the control group at 12 months

This hypothesis will be assessed with a 2 (group) × 2 (control) chi-square analyses as there is no time factor in these analyses. However, we will use multilevel logistic regression to control for the nesting of patients within clinics. BP control will be defined as occurring when systolic BP < 140 mm Hg and diastolic BP < 90 mm Hg at 12 months. Effect sizes will be reported as odds ratios with 95% CI. The covariates describe above will be included in the analyses as needed. For those participants without BP data at 12 months, we will estimate their BP control using data from previous time points.

#### Secondary hypothesis two: patients in the intervention group will have higher levels of physical activity, greater weight loss, and intake of fruits and vegetables than those in the control group at 12 months

Similar to the primary hypothesis testing, we will perform separate multilevel MANOVAs (observations within person within health center) with one within-patient factor (Time) and one between-patient factor (Group) for the summary measure of physical activity, weight, and the summary measure of intake of fruits and vegetables. For each analysis, we will allow for group differences in the variance of the outcome at follow-up and the serial correlation of baseline with follow-up assessments. Because changes in weight tend to be proportional to baseline weight, we will follow the widely used strategy of analysing the percent change in weight; thus, there will be no within-patient component to this analysis per se, but we will allow for group differences in the SD of percent change. Similar to the analysis for the primary hypothesis, if evidence of selection or attrition biases or group differences resulting from randomization is detected, predicted values of the corresponding probit analysis will be incorporated as covariates into these analyses. However, we may consider introducing an adjustment to protect against the experiment-wide risk of a Type I error. If so, we would use Holm’s modified Bonferroni procedure, which is less conservative than the traditional Bonferroni procedure, but still controls for the experiment-wide α-level [39].

#### Secondary hypothesis three: patients in the intervention group will maintain higher BP control rates and greater reduction of both SBP and DBP than those in the control group at 24 months

These analyses will be identical to those for the primary hypothesis except that an additional 24-month time point will be added to the multilevel model. We will consider a spline regression model to assess the possibility that there will be no further linear decrease in BP in the groups, but simply a maintenance of the changes achieved at 12 months.

### Trial status

The study began in November 2012 and to date, we have recruited and randomized all 32 community health centers. Patient recruitment is ongoing with about 60% of the patients recruited to date. Follow up is almost completed for the first cohort of eight sites with a total of 190 patients; and the final cohort of eight sites was randomized in January 2014.

## Discussion

Although the prevalence of hypertension and its related complications are well documented in SSA [40], few investigators have evaluated the effectiveness of interventions targeted at BP control and CV risk in the region. Fortunately, the WHO has undertaken extremely vital programs and provided a roadmap, and an agenda to tackle the hypertension crisis in LMIC, including SSA [15]. One such program with respect to CV risk-reduction is the strategy of task-shifting designed to address the multiple layers of the CVD epidemic including screening, counselling on lifestyle modification, initiation of treatment, and referral to specialist care with the use of community health workers [25]. Although the reliability of having community health nurses deliver the WHO strategy for CV risk assessment and optimal hypertension control when compared to ‘expert’ physicians in primary care settings has been established in several LMIC countries [41], its implementation is almost non-existent in SSA. More importantly, in order for task-shifting strategies to be considered effective, evidence of its implementation for addressing the CVD epidemic as part of existing healthcare systems in LMICs are paramount. Such studies integrated into existing healthcare systems will guarantee subsequent adoption of interventions if proven successful.

This study protocol addresses the gap in the literature by evaluating implementation of the WHO Package in community health centers in Ghana as part of the Ghana Health Service—the major healthcare organization in the country. Findings from this study, if successful, will be important for several reasons. First, majority of the population in SSA receive their care in community health centers and are largely unable to afford care at the secondary and tertiary care centers due to very high out-of-pocket payments and medication costs [42]. Second, the effectiveness of the WHO Package in primary healthcare centers in LMICs has been successfully evaluated making it ripe for implementation and potential dissemination [10,41]. Third, a recent survey of primary healthcare centers with respect to management of hypertension, suggests that significant proportion of community health workers initiated thiazide diuretics as first-line therapy for hypertensive patients despite low knowledge of hypertension treatment guidelines, thus supporting the need to enhance this skill and its implementation at the primary care level [28]. In the same survey, about two-thirds of hypertensive patients utilized primary healthcare centers both for diagnosis and for follow-up care. This implies that primary healthcare centers have the potential to contribute significantly to management of CVD, and that targeting hypertension control at the primary care level should yield better CV outcomes than population-based approaches. Finally, this clinical trial addresses important systems-level barriers to optimal hypertension control in SSA. Although barriers to optimal hypertension control exists at the level of the patients, providers, and the healthcare systems [22], in SSA countries, systems-level barriers seem to have more adverse impact on healthcare in general, particularly among those with chronic diseases [28]. In this regard, lack of access to care (particularly among the poor), suboptimal staffing of healthcare facilities, and limited capacity for laboratory investigations that complement CV risk assessment are major barriers limiting the capacity of countries in SSA to manage CVD at the primary care level [9]. This situation is made worse by the brain drain that has plagued SSA for over 20 years [12,14]; with a resultant acute shortage of health workers available to implement primary and secondary CVD prevention at the primary care level. Task shifting can be effective in mitigating this burden, particularly among hypertensive patients.

If findings from the proposed study are successful, this will provide the evidence-base for policy makers to recommend similar strategies across healthcare systems with respect to comprehensive CV risk reduction and hypertension control in resource-poor settings because several SSA countries like Tanzania, Kenya, Cameroon, and Nigeria have already implemented a healthcare system that has at its hub the use of community health workers to deliver primary care, making generalizability of the proposed task-shifting strategy very feasible [42]. In Nigeria, for example, non-physician healthcare providers including public health nurses, nurse/midwives, and community health workers are responsible for managing primary healthcare facilities, which function as outpatient clinics, while physicians manage secondary and tertiary healthcare facilities. Non-physician healthcare providers are typically trained in nursing/midwifery or community health extension work for a period ranging from two to four years [43]. Similarly, national health insurance scheme has been instituted in Nigeria and assessment of its effectiveness in improving BP control in the uninsured patient would also provide the impetus for other SSA countries to adopt this strategy, if proven successful.

## Conclusions

Ghana and other countries in SSA are experiencing an epidemic of CVD propelled by rapidly increasing rates of hypertension. Socioeconomic barriers, lack of insurance coverage, and shortage of physicians limit the capacity of SSA countries to implement CVD prevention. Task shifting of primary care duties from physicians to non-physician health care providers is a potentially cost-effective strategy for mitigating systems-level barriers to optimal hypertension control in SSA. In this regard, the WHO developed and successfully evaluated the effectiveness of a WHO Package targeted at CV risk assessment and hypertension control, delivered by community health nurses (CHNs) in low resource settings. However widespread implementation of the WHO Package has not been evaluated in SSA. The availability in Ghana of national health insurance scheme for uninsured patients, and widespread implementation of CHPS program that uses CHNs for delivery of primary care services, presents a unique opportunity to evaluate the impact of both strategies on hypertension control. Thus, this study will evaluate the comparative effectiveness of the WHO CVD risk management package for hypertension control delivered by CHNs as part of Ghana’s CHPS program plus provision of health insurance coverage, versus provision of health insurance coverage alone, on BP reduction among 640 patients who receive care in 32 CHCs in Ghana. Findings from this study will provide policy makers and other stakeholders needed information to recommend scalable and cost-effective policy with respect to comprehensive CV risk reduction and hypertension control in resource-poor settings.

## Abbreviations

SSA: Sub-Saharan Africa; CVD: Cardiovascular diseases; WHO: World Health Organization; CHC: Community health center; CHPS: Community-based health planning and services; IG: Intervention group; CG: Control group; LMICs: Low and middle-income countries; NPHCP: Non-physician Healthcare Providers; NHIS: National health insurance scheme; BP: Blood pressure; CCB: Calcium channel blocker; BB: Beta blocker; ACE: Angiotensin converting enzyme inhibitor; ICC: Intraclass correlation coefficient.

## Competing interests

The authors declare that they have no actual or potential competing interests.

## Authors’ contributions

GO conceived of the study design and drafted the manuscript. JPR made substantial contributions to study design and manuscript preparation. JG participated in the coordination of the study and manuscript preparation. WC programmed the randomization procedure, and manuscript preparation. KA and KK participated in the data acquisition and manuscript preparation. MN assisted with the study coordination and manuscript preparation. RC contributed to the study design and manuscript preparation. All authors have read and approved the final manuscript.
